# Identification of a PAK6-Mediated MDM2/p21 Axis That Modulates Survival and Cell Cycle Control of Drug-Resistant Stem/Progenitor Cells in Chronic Myeloid Leukemia

**DOI:** 10.3390/ijms26136533

**Published:** 2025-07-07

**Authors:** Andrew Wu, Min Chen, Athena Phoa, Zesong Yang, Donna L. Forrest, Xiaoyan Jiang

**Affiliations:** 1 Collings Stevens Chronic Leukemia Research Laboratory, Terry Fox Laboratory, British Columbia Cancer Research Institute, Vancouver, BC V5Z 1L3, Canadamchen@bccrc.ca (M.C.);; 2 Department of Medicine, University of British Columbia, Vancouver, BC V6T 1Z4, Canada; 3 Department of Hematology, The First Affiliated Hospital of Chongqing Medical University, Chongqing 400000, China; 4 Leukemia/Bone Marrow Transplant Program of British Columbia, Vancouver, BC V5Z 1M9, Canada; 5 Department of Medical Genetics, University of British Columbia; Vancouver, BC V6T 1Z4, Canada

**Keywords:** CML, leukemic stem cells, TKI therapy, TKI resistance, cell cycle, Imatinib, PAK6, small molecule inhibitors, senescence, DNA damage

## Abstract

Chronic myeloid leukemia (CML) is a leading example of a malignancy where a molecular targeted therapy revolutionized treatment but has rarely led to cures. Overcoming tyrosine kinase inhibitor (TKI) drug resistance remains a challenge in the treatment of CML. We have recently identified *miR-185* as a predictive biomarker where reduced expression in CD34^+^ treatment-naïve CML cells was associated with TKI resistance. We have also identified *PAK6* as a target gene of *miR-185* that was upregulated in CD34^+^ TKI-nonresponder cells. However, its role in regulating TKI resistance remains largely unknown. In this study, we specifically targeted *PAK6* in imatinib (IM)-resistant cells and CD34^+^ stem/progenitor cells from IM-nonresponders using a lentiviral-mediated *PAK6* knockdown strategy. Interestingly, the genetic and pharmacological suppression of PAK6 significantly reduced proliferation and increased apoptosis in TKI-resistant cells. Cell survivability was further diminished when IM was combined with PAK6 knockdown. Importantly, PAK6 inhibition in TKI-resistant cells induced cell cycle arrest in the G2-M phase and cellular senescence, accompanied by increased levels of DNA damage-associated senescence markers. Mechanically, we identified a PAK6-mediated MDM2-p21 axis that regulates cell cycle arrest and senescence. Thus, PAK6 plays a critical role in determining alternative cell fates in leukemic cells, and targeting PAK6 may offer a therapeutic strategy to selectively eradicate TKI-resistant cells.

## 1. Introduction

Chronic myeloid leukemia (CML) is a myeloproliferative disorder characterized by the t(9;22)(q34;q11) chromosomal translocation, which results in the constitutively active *BCR::ABL1* fusion oncogene [1]. Although CML is successfully managed by tyrosine kinase inhibitors (TKIs) for the most part, the disease remains elusive to curative therapy, as patients who respond to therapy often receive continuous treatment or risk relapse due to the persistence of leukemic stem cells (LSCs) [2,3,4]. In addition, several drug resistance mechanisms also contribute to a barrier to TKI response, leading to the study and development of combination strategies that simultaneously target BCR::ABL1 and these alternative oncogenic pathways [5,6,7,8]. We have recently identified *p21-activated kinase 6* (*PAK6*) as a target gene of *miR-185*, a microRNA suppressed in TKI-resistant stem/progenitor cells, which is also a predictive biomarker for TKI response in CML patients treated with imatinib (IM) or nilotinib [9,10,11,12]. However, its precise role in regulating TKI resistance remains largely unknown.

The p21-activated kinases (PAKs) are a family of serine/threonine kinases that regulate essential biological processes such as cytoskeletal dynamics, cell survival, and proliferation [11,13,14,15]. The six PAK family members are divided into two groups based on their structure and activation mechanisms. Group I PAKs (PAK1-3) have an autoinhibitory domain (AID) that suppresses their kinase activity until the binding of Cdc42/Rac GTPases induces a conformational change [13,15]. In contrast, Group II PAKs (PAK4-6) lack a well-defined AID and are constitutively phosphorylated but are regulated by pseudosubstrate regions that bind the kinase domain [13,15]. Dysregulation of PAKs is frequently observed in human cancers [11,14].

PAK6 has been known to play a prevalent role in promoting several solid tumors, including prostate, gastric, and colorectal cancers [14,16]. Although some group I PAKs, such as PAK1 and PAK2, have been identified as therapeutic targets in hematologic malignancies, little is understood about the role of PAK6, especially in the context of CML [17,18,19]. Interestingly, *PAK6* was more highly expressed in CD34^+^ CML cells compared to healthy bone marrow samples and in TKI-nonresponder patients compared to responders [12]. PAK6 was also elevated in the more primitive CD34^+^CD38^−^ compartment relative to the differentiated CD34^−^ compartment [12].

In this study, we investigated the role of PAK6 in mediating TKI resistance and cell survival using the pan-PAK inhibitor PF-3758309, as well as a lentiviral-mediated *PAK6* knockdown strategy, in IM-resistant cell lines and CD34^+^ TKI-nonresponder cells. We find that pharmacological and genetic suppression of PAK6 reduces survivability and proliferation, particularly when combined with IM treatment. We also demonstrate that PAK6 knockdown can induce DNA damage-associated G2-M cell cycle arrest and senescence by modulating the expression of MDM2, p21, and other senescence protein markers. Thus, our findings suggest that PAK6 may be an attractive therapeutic target due to its role in mediating the DNA damage response and governing multiple cell fates, including senescence and apoptosis, in the context of CML.

## 2. Results

### 2.1. Lentiviral-Mediated PAK6 Knockdown Sensitizes TKI-Resistant CML Cells to IM

**Figure 1:**
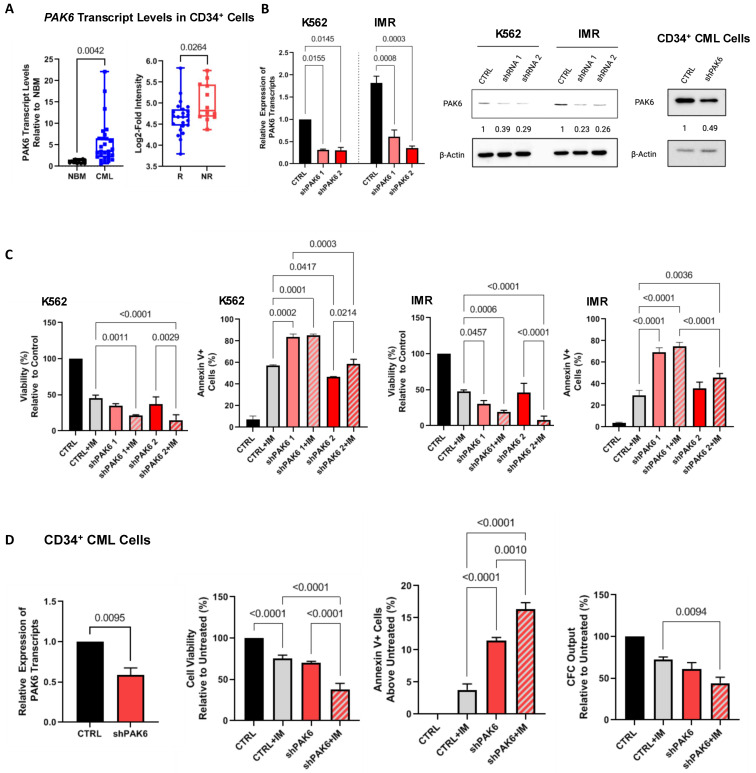
**Knockdown of PAK6 inhibits cell survival and proliferation.** (**A**) qRT-PCR analysis of *PAK6* transcript levels in CD34^+^ stem/progenitor cells from 23 CML samples compared to 11 normal bone marrow (NBM) samples (**left**) and analysis of the GSE14671 microarray dataset of CD34^+^ BM samples from 24 IM-responders and 12 IM-nonresponders (**right**). (**B**) Western blot analysis of knockdown of PAK6 with two shRNA constructs in K562 and IMR cells and CD34^+^ primary CML cells, quantifying protein expression levels relative to β-Actin as indicated. (**C**) Viability and apoptosis assays were conducted in PAK6 knockdown cells with or without IM. (**D**) qRT-PCR analysis of *PAK6* transcript levels in CD34^+^ nonresponder cells with PAK6 knockdown (n = 3). Viability, apoptosis, and CFC assays were conducted in these PAK6 knockdown cells with or without IM.

We have previously shown that *PAK6* mRNA transcript levels are highly expressed in CD34^+^ CML cells, including IM-nonresponder patients, compared to IM-responder patients [12]. We validated this observation in CD34^+^ cells from an additional 23 CML patient samples and 11 normal bone marrow (NBM) samples by qRT-PCR (Table S1) as well as by analyzing the GSE14671 microarray dataset of CD34^+^ BM samples from 24 IM-responders and 12 IM-nonresponders. Indeed, *PAK6* gene expression was significantly higher in CML compared to NBM (*p* = 0.0042) and in the microarray set of IM-nonresponders compared to IM-responders (*p* = 0.0264, Figure 1A) [20].

To explore the specific role of PAK6 in mediating TKI resistance in CML, we generated a lentiviral-mediated shRNA knockdown system with two constructs targeting different sequences of PAK6 and demonstrated that both specifically suppressed PAK6 gene expression at both the RNA and protein levels (up to 80%, Figure 1A and Figure S1A). Interestingly, PAK6 knockdown alone significantly decreased viability and increased apoptosis more than IM in K562- and K562-resistant (IMR) cell lines in vitro (*p* < 0.045, Figure 1C). The addition of IM combined with the PAK6 knockdown had an increased additive effect (*p* < 0.0214). These results support our findings from a previous study using the pan-PAK inhibitor PF-3758309 alone or in combination with TKIs to suppress CML cell survivability [12].

We then knocked down PAK6 in CD34^+^ stem/progenitor cells from IM-nonresponder patient samples (n = 3) to determine if we could inhibit survivability to a similar degree. Although PAK6 mRNA transcript and protein expression were reduced by 50% in these cells, we found that PAK6 knockdown could indeed reduce cell viability and induce apoptosis (*p* < 0.0095, Figure 1B,D). Additionally, combining IM with PAK6 knockdown in these cells could significantly enhance TKI-mediated killing compared to either treatment alone (*p* < 0.001). Colony-forming cell (CFC) assays also showed that combining IM and PAK6 knockdown inhibited CFC output over single treatment conditions (*p* = 0.0094, Figure 1D). The results of these survival assays, especially in CD34^+^ stem/progenitor cells, suggest that PAK6 knockdown re-sensitizes resistant CML cells to TKI-mediated killing effects.

### 2.2. Lentiviral-Mediated PAK6 Knockdown Induces G2/M Cell Cycle Arrest in IM-Resistant Cells

**Figure 2:**
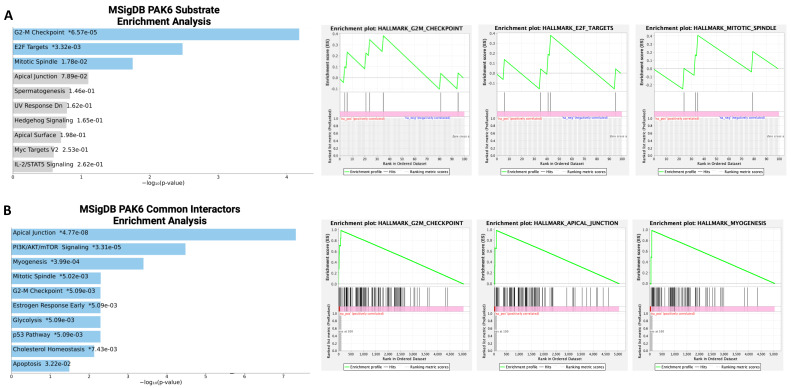
**Bioinformatics analysis of PAK6 substrates and common protein interactors.** (**A**,**B**) Gene set enrichment analyses (GSEAs) of PAK6 substrates and common protein interactors identified from the Harmonizome 3.0 dataset repository revealed significant enrichment of biological processes related to the G2-M checkpoint. * = *p* value (**left**). Green line indicate enrichment profile and gray lines indicate raking metric scores (**right**).

**Figure 3:**
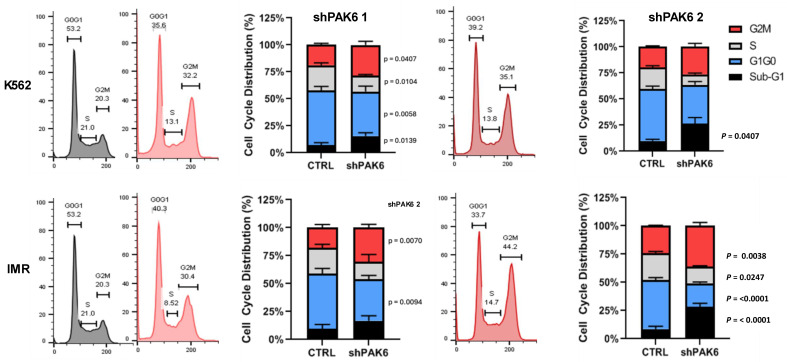
**Lentiviral-mediated PAK6 knockdown induces G2/M cell cycle arrest in IM-resistant cells.** Flow cytometry analysis of cell cycle distribution in K562 and IM-resistant cells (IMR) following knockdown of PAK6 with two shRNA constructs. PAK6 knockdown reduced the number of cells in the G0/G1 and S phases and induced the accumulation of cells in the G2-M phases compared to the control.

Since we demonstrated that PAK6 suppression can inhibit CML cell proliferation and survival, we wanted to investigate additional oncogenic mechanisms governed by PAK6 in CML. To uncover the biological processes involved downstream of PAK6, we first compiled gene sets of known and predicted PAK6 substrates, as well as common protein interactors, from the Harmonizome 3.0 dataset repository and ran gene set enrichment analyses (GSEAs) using the Enrichr platform [21]. For both the PAK6 substrate and common protein interactor gene sets, we noticed the G2-M checkpoint biological processes to be significantly enriched (Figure 2A,B, Table S3). However, since the GSEA was performed using gene sets largely curated through mass spectrometry, synthetic peptides, and in vitro kinase experiments in non-leukemic cells, we then conducted cell cycle assays on K562 and IMR cells following PAK6 knockdown to investigate changes in cell cycle dynamics in the context of CML. In support of the enrichment analysis, we found that PAK6 knockdown reduced the number of cells in the G0/G1 (*p* = 0.0058) and S (*p* = 0.0104) compartments and significantly induced accumulation of cells in the G2-M compartment in K562 cells transduced with the two different PAK6 knockdown constructs as compared to the control (up to 35% vs. 20%, *p* = 0.0407, Figure 3). Interestingly, this arrest of cells in the G2-M compartment was greater in IMR cells, showing that IMR cells may experience more sensitivity to PAK6 knockdown compared to K562 cells (up to 44%, *p* = 0.0038, Figure 3). This finding suggests that PAK6 plays a role in facilitating leukemia growth and progression by dysregulating G2-M checkpoint controls.

### 2.3. Identification of an MDM2-P21 Axis Mediated by PAK6 in IM-Resistant Cells

**Figure 4:**
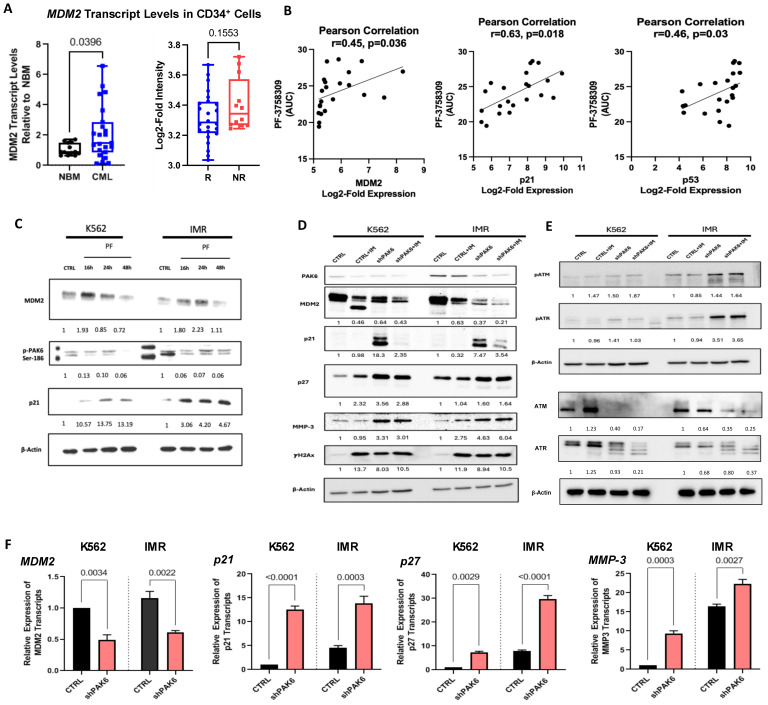
**Identification of a PAK6-mediated MDM2-P21 axis in CML cells.** (**A**) qRT-PCR analysis of *MDM2* transcript levels in CD34^+^ cells from 23 CML samples compared to 11 NBM samples (**left**) and analysis of the GSE14671 microarray dataset from 24 IM-responders and 12 IM-nonresponders (**right**). (**B**) Correlation analysis using the Cancer Therapeutics Research Portal (CTRPv2) database between *MDM2*, *p21*, and *p53* expression and their sensitivities to the PAK inhibitor PF-3758309, as measured by area-under-curve (AUC) values. (**C**) Western blot analysis of PAK6 phosphorylation, MDM2, and p21 protein expression in K562 and IM-resistant cells (IMR) treated with PF-3758309 (PF) at 16, 24, and 48 h. Quantification of protein expression levels relative to β-Actin as indicated. (**D**–**E**) Western blot analysis of protein expression and phosphorylation of senescence-associated biomarkers and DNA damage markers, following PAK6 knockdown in K562 and IMR cells, quantifying protein expression levels relative to β-Actin as indicated. (**F**) qRT-PCR analysis of transcript levels of senescence-associated biomarkers in PAK6 knockdown cells.

Given that PAK6 knockdown could induce G2-M cell cycle arrest, we explored the molecular pathways mediated by PAK6 to dysregulate the cell cycle by searching PAK6 substrates identified from in vitro kinase studies curated by the PhosphoSitePlus platform, one of which was MDM2, a key negative regulator of p53 and cell cycle checkpoint blocker p21 (Figure S1B) [22]. Interestingly, we demonstrated that *MDM2* mRNA transcript levels were highly elevated in CD34^+^ stem/progenitor cells from 23 CML samples as compared to 11 NBM samples by qRT-PCR analysis (*p* = 0.0396, Figure 4A). This is further supported by showing increased *MDM2* expression in IM-nonresponders as compared to IM-responders by analyzing *MDM2* expression in the GSE14671 microarray dataset (Figure 4A) [20]. There were no noticeable differences in gene expression of the other PAK6 substrates between these two groups (Figure S1C). We then conducted univariate and correlation analyses on leukemic cell line and PAK inhibitor (PF-3758309) drug activity data from the Cancer Therapeutics Research Portal Version 2 (CTRPv2) database using the CellMinerCDB analysis tool [23]. As expected, there was a positive correlation (r = 0.45, *p* = 0.036) between *MDM2* expression and PF-3758309 area-under-curve (AUC) values, indicating that cells with higher expression of *MDM2* were more susceptible to the PAK inhibitor (Figure 4B). This was supported by positive correlations with *p21* and *p53* (Figure 4B). Indeed, we demonstrated a time-dependent reduction in MDM2 total protein levels following inhibition of PAK6 phosphorylation by PF-3758309 in K562 and IMR cells (Figure 4C). Interestingly, we also found corresponding increases in p21 protein expression that appear to be induced in a p53-independent manner, as the K562 cell line is known to be p53-null (Figure 4C). In our PAK6 knockdown models, we observed similar protein dynamics where MDM2 is reduced and p21 is significantly increased in response to PAK6 knockdown (Figure 4D). We also observed that treating PAK6 knockdown cells with IM could further inhibit MDM2 protein levels. Notably, IM treatment in PAK6 knockdown cells could reverse the induction of p21 (Figure 4D). Altogether, it appears that the G2-M cell cycle arrest we observed following PAK6 knockdown in CML cells may result from the induction of p21 protein expression mediated by MDM2.

### 2.4. Pharmacological and Genetic PAK6 Suppression Induces Senescence in IM-Resistant Cells

**Figure 5:**
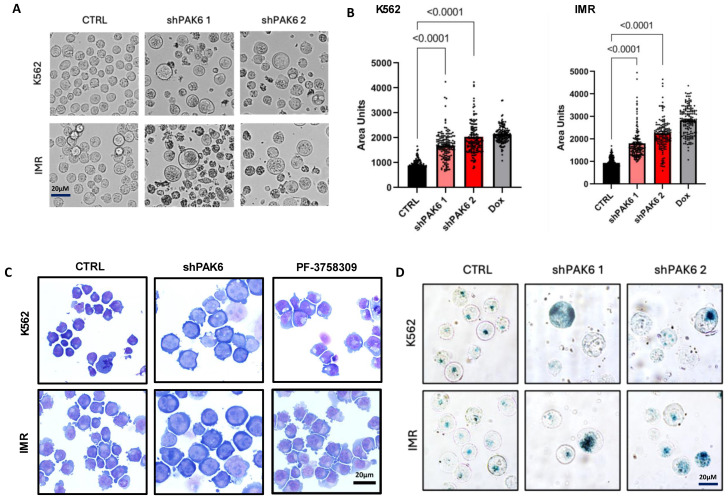
**Inhibition of PAK6 increases cell size in CML cells.** (**A**,**B**) Representative phase-contrast images and quantification of cell size in K562 and IM-resistant cells (IMR) following PAK6 knockdown, including senescence-inducing compound doxorubicin (Dox), used as a positive control. (**C**) GIEMSA staining of K562 and IMR cells following PAK6 knockdown or treatment with PF-3758309 (10 nM). (**D**) Representative images of senescence-associated β-galactosidase (SABG) staining of K562 and IMR cells following PAK6 knockdown.

**Figure 6:**
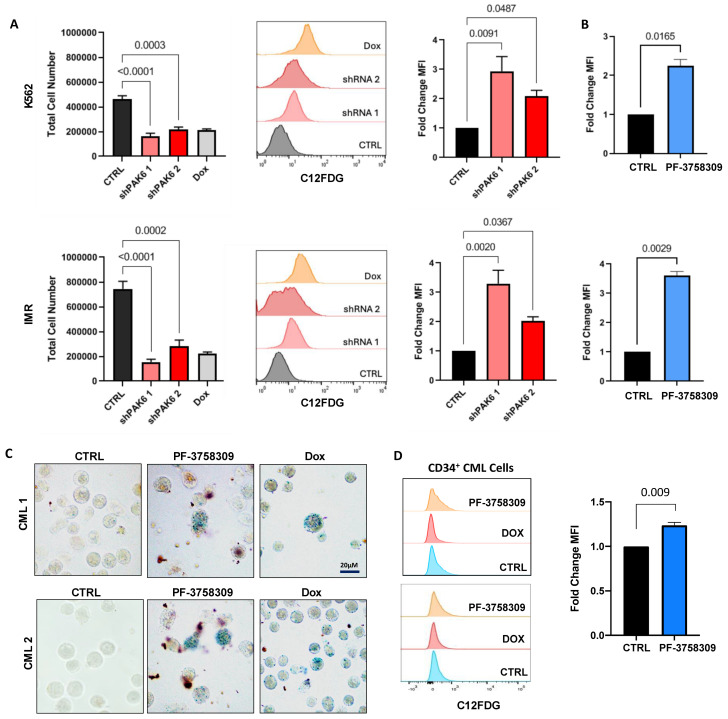
**PAK6 knockdown induces senescence-associated phenotypes in CML cells.** (**A**) Total cell numbers generated in K562 and IM-resistant cells (IMR) following PAK6 knockdown, including a Dox control, after 48 h of treatment with C12FDG. C12FDG-mediated SABG assays were then performed on these cells and analyzed by FACS histograms, with corresponding quantification of mean fluorescence intensity MFI (bar plots). (**B**) Fold changes of differences in K562 and IMR cells treated with the PAK inhibitor PF-3758309 (10 nM), with corresponding quantification of MFI by C12FDG-mediated SABG assays. (**C**,**D**) Representative images of SABG staining and C12FDG-mediated SABG assays conducted in CD34^+^ nonresponder primary cells treated with PF-3758309 (10 nM). Intense staining of blue color in cells indicates increased β-galactosidase expression.

It has been well established that p21 is not just a key regulator of cell cycle checkpoints but also a marker for senescence. Cellular senescence is traditionally defined as a state of permanent cell cycle arrest and is considered an alternative cell fate to apoptosis. With PAK6 knockdown already shown to cause G2-M cell cycle arrest, we investigated if this arrest was related to senescence. However, there is a consensus that senescence cannot be determined by a single marker alone and that several senescence-associated phenotypes must be observed for confirmation. One such phenotype is an increase in cell size and morphology. Interestingly, quantification of phase-contrast images and GIEMSA staining of K562 and IMR cells following PAK6 knockdown or treatment with a PAK inhibitor revealed a two-fold increase in cell size compared to control cells transduced with a control vector (*p* < 0.0001, Figure 5A–C). This enlargement of cells was similar to that induced by doxorubicin, a senescence-inducing compound serving as a positive control (Figure 5B). Senescent cells are also known to accumulate lysosomal β-galactosidase, which can be detected by senescence-associated β-galactosidase (SABG) staining. K562 and IMR cells with PAK6 knockdown placed on Poly-L-Lysine slides and stained for SABG showed a more intense blue color resulting from the cleavage of X-Gal due to increased β-galactosidase expression (Figure 5D). To validate these results, these PAK6 knockdown cells were analyzed with a more sensitive C12FDG-mediated SABG assay by FACS. As expected, the total cell number did not increase in K562 (*p* < 0.0003) and IMR (*p* < 0.0002) PAK6 knockdown cells compared to controls over 48 h of treatment with C12FDG (Figure 6A). FACS analysis revealed a three-fold increase in C12FDG fluorescence following PAK6 knockdown in both K562 (*p* < 0.0487) and IMR cells (*p* < 0.0367, Figure 6A). This result was further supported by observation of increased senescence cells by treatment of the PAK inhibitor PF-3758309 in both K562 and IMR cells (*p* < 0.0165, Figure 6B).

Interestingly, we also found that senescence could be induced by modulation of the MDM2-p21 axis following PAK6 suppression in CD34^+^ IM-nonresponder cells. On its own, we have already demonstrated that PF-3758309 increased apoptosis in TKI-insensitive CML cells. Here, we observed that treatment of PF-3758309 in CD34^+^ IM-nonresponder cells could induce senescence, as evidenced by increased chromogenic and FACS-mediated SABG staining (*p* = 0.0090, Figure 6C,D).

### 2.5. Suppression of PAK6 Induces Activation of Senescence-Associated Protein Markers in IM-Resistant Cells

We then investigated molecular changes in several senescence-associated protein biomarkers in response to PAK6 knockdown at both RNA and protein levels. Aside from p21 induction, as described earlier, p27, MMP3, and the DNA damage marker γH2Ax were significantly increased (Figure 4D,F). To further explore the effect of PAK6 aberration on DNA damage, we also probed for and found increased phosphorylation of ATM and ATR, which are key players and additional markers of DNA Damage Response (DDR), in cells with PAK6 knockdown alone and in combination with IM (Figure 4E). We also noticed an associated decrease in total ATM and ATR levels in these conditions. Notably, some of these protein phosphorylation and expression changes were more obvious in IM-resistant cells than in the control cells. Additional IM treatment did not enhance senescence and DDR-associated protein expression beyond that achieved with PAK6 knockdown alone.

**Figure 7:**
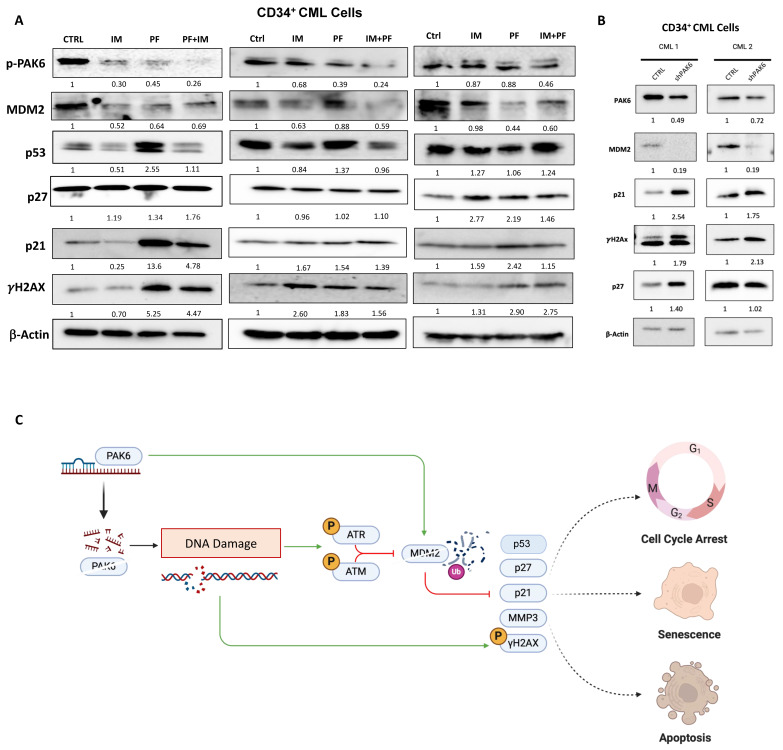
**PAK6 inhibition elevates senescence-associated and DNA damage response biomarkers in CD34^+^ CML cells.** (**A**) Western blot analysis of protein expression of senescence-associated biomarkers, including p21, p27, MMP3, and the DNA damage marker γH2Ax, following IM (5µM) or PF-3758309 (10 nM), alone or in combination in CD34^+^ CML cells (n = 3). (**B**) Western blot analysis of these proteins in CD34^+^ CML cells with lentiviral-mediated PAK6 knockdown (n = 2). Quantification of protein expression levels relative to β-Actin as indicated. (**C**) Model of the role of PAK6 in cell cycle progression, senescence, and DNA damage response in CML. PAK6 promotes leukemia growth by regulating the MDM2-p21 axis, facilitating cell cycle progression, and suppressing senescence. PAK6 knockdown disrupts this axis, leading to G2-M cell cycle arrest, induction of senescence, and activation of the DNA damage response.

Importantly, we observed changes in senescence-associated protein markers within the MDM2-p21 axis following pharmacologic and genetic PAK6 inhibition in CD34^+^ IM-nonresponder cells (n = 3, Figure 7A). In response to PF-3758309 treatment, we observed a significant decrease in MDM2 protein levels and increases in p53, p21, and γH2Ax (Figure 7A). Interestingly, in both cell line and primary cell models of PAK6 inhibition, treatment of IM in PAK6 knockdown cells decreased the expression of p21 induced by PAK6 knockdown. In primary cells where p53 was also induced following PF-3758309 treatment, the addition of IM also negated p53 elevation (Figure 7A). These observations were further supported by lentiviral-mediated PAK6 knockdown in CD34^+^ CML cells, which led to a decrease in MDM2 as well as associated increases in p21, p27, and γH2Ax, which was reflective of what we observed in our cell line knockdown models (Figure 7B). Altogether, this suggests a possible dynamic interplay between senescence and apoptosis cell fates (Figure 7C).

## 3. Discussion

The development of TKIs has significantly improved the treatment of CML. However, TKI therapy is not curative, and patients often require lifelong treatment due to potential relapse from drug resistance and activation of BCR::ABL1-independent pathways. We recently identified PAK6 as a target gene of miR-185 in CD34^+^ TKI-nonresponder cells and demonstrated that the use of a pan-PAK inhibitor, PF-3758309, reduced cell growth in TKI-nonresponder CD34^+^ stem/progenitor cells [12,24]. However, molecular mechanisms of PAK6-mediated drug resistance are largely unknown; in particular, PF-3758309 could reduce mitochondrial staining and ROS production in TKI-nonresponder CD34^+^ CML cells, indicating metabolic effects that require further investigation [12].

To demonstrate that the observed effects by PF-3758309 were specifically due to PAK6 inhibition, we performed shRNA-mediated PAK6 knockdown. Indeed, in our IM-resistant cell line model and CD34^+^ nonresponder patient cells, PAK6 knockdown or pharmacological suppression reduced cell viability, increased apoptosis, and inhibited colony formation in TKI-resistant cells. We then found through gene set enrichment analysis that PAK6 substrates and common protein interactors were heavily involved in the G2-M checkpoint, and we demonstrated that PAK6 knockdown in IM-resistant cells induced G2-M cell cycle arrest [25,26,27]. To uncover the mechanisms behind PAK6 and cell cycle arrest, we evaluated MDM2 from a list of known PAK6 substrates from PhosphoSitePlus due to its crucial role as a negative regulator of p53 and the cell cycle checkpoint inhibitor p21 [22,28,29]. Others have previously shown that PAK6 phosphorylates MDM2 at the Ser186 and Thr158 sites, activating MDM2 to ubiquitinate the androgen receptor and inhibit tumor growth in prostate cancer cells [30]. In contrast to our previous study, where we established *PAK6* as an oncogene targeted by the *miR-185* tumor suppressor, this study suggests that PAK6 may have tumor-suppressive functions in prostate cancer.

In CML, it has been shown that *MDM2* is more highly expressed in quiescent CD34^+^ CML progenitor cells compared to their proliferating counterparts and that MDM2 plays a vital role in mediating TKI resistance in CML. Inhibition of MDM2 has been demonstrated to sensitize TKI-resistant CD34^+^CD38^-^ proliferating and quiescent cells to nilotinib [31]. In our patient samples, we also found that *MDM2* transcript expression was higher in CML patients than in those with NBM. Microarray data from the GSE14671 dataset showed increased expression in IM-nonresponder samples compared to IM-responders [20]. However, we noticed in both our own and the GSE14671 studies that the expression of *MDM2* in CML patient cells appeared to be quite heterogeneous. In addition, univariate and correlation analyses of leukemic cell line data and PF-3758309 activity, conducted through CellMinderCDB, revealed that cells with higher expression of *MDM2* were more sensitive to PF-3758309 [23,32]. In this study, we demonstrate for the first time that PAK6 suppression can indeed modulate the MDM2-p21 axis in CML cells. At the basal level, MDM2 is strongly expressed in both K562 and IMR cells, whereas p21 is minimally expressed. However, PF-3758309 and PAK6 knockdown induced a corresponding decrease in MDM2 total protein expression and a significant increase in p21 protein expression. Canonically, MDM2 is understood to be an E3 ubiquitin ligase that negatively regulates p53 by tagging it for ubiquitination [28,29,33]. As p21 is downstream of p53, reduction in p53 therefore upregulates p21 activity. Interestingly, in K562 and IMR cells, the upregulation of p21 following PAK6 is independent of p53, as these cells are known to have mutational inactivation of the *p53* gene and therefore do not express the p53 protein [34]. The existing literature has suggested that MDM2 may directly inhibit p21 independent of p53 and ubiquitination through proteasome-mediated degradation in PC3 prostate cancer cells [35]. While this may also be a possible mechanism of p21 regulation in K562 and IMR cells, further work is required for confirmation. Indeed, it is critical to determine if a physical interaction between PAK6 and MDM2 or p21 is involved in understanding the molecular mechanisms by which this pathway mediates protein phosphorylation and protein expression of key proteins, as well as their biological outcomes, through the knockdown or overexpression of these proteins or their mutants. In particular, it would be interesting to determine if PAK6 can directly interact with a specific domain or phosphorylate several sites of MDM2 or p21 in drug-resistant cells, as well as mediate protein ubiquitination and degradation.

Furthermore, CD34^+^ stem/progenitor cells from primary patient samples, unlike K562 cells, can be p53 positive. Previous work on human diploid fibroblasts has shown that the degree of *p53* expression can influence whether a cell undergoes senescence or apoptosis [36]. In our hands, CD34^+^ CML cells exhibited increased p53 levels following PAK6 inhibition by PF-3758309, which subsequently led to an increase in p21. Since PAK6 inhibition can induce a corresponding decrease in MDM2 protein expression and a significant increase in p21 protein expression in the presence of p53 in CD34^+^ CML patient cells, a similar observation was made in our cell line model system without expression of p53, suggesting that the activity of the MDM2-p21 axis is mainly independent of p53. It possibly induces ubiquitination through proteasome-mediated protein degradation in these cells. However, the MDM2-p53 interaction is a crucial regulatory mechanism in cancer cells; this particular interaction may also contribute to the biological and molecular changes observed in CD34^+^ CML cells under PAK6 inhibition. Indeed, further work is required to elucidate how the presence or absence of p53 affects PAK6 knockdown-associated phenotypes in the context of CML [31]. P21 upregulation in response to PAK6 knockdown also contradicts previous findings in prostate cancer cells, where PAK6 knockdown induced expression of cyclin D1, a proliferation marker negatively regulated by p21, enhancing tumor growth in vitro and in vivo [30]. This suggests that PAK6-mediated regulation of MDM2 may exert both oncogenic and tumor-suppressive effects depending on the cancer context. Nevertheless, our findings support our previous work characterizing *PAK6* as an oncogene that facilitates cell cycle progression and survival in CML.

PAK6 knockdown also induced senescence-associated phenotypes like enlarged cell size, increased SABG staining, and elevation of senescence protein markers, including p21. PAK6 knockdown showed increased expression of p27, γH2AX, and MMP3. Like p21, p27 is also a tumor suppressor and is implicated in G2-M cell cycle arrest [37]. Interestingly, the induction of p21, p27, and γH2AX also suggests that G2-M arrest and senescence in PAK6 knockdown cells may be part of the DNA Damage Response (DDR) [38,39,40]. It has been demonstrated that p21 is involved in G2 arrest of the cell cycle in p53-deficient DLD1 colon cancer cells following DNA damage [41]. Additionally, p27-deficient mice have been observed to have impaired G2-M arrest in response to DNA damage [37]. Recently, it has been revealed that PAK6 enhances gastric cancer chemoresistance by modulating the DDR [16]. Specifically, PAK6 activates the ATR cascade, which includes CHK1, and promotes homologous recombination [16]. Interestingly, we found that PAK6 knockdown cells had increased phosphorylated levels of ATM and ATR, indicating that PAK6 knockdown possibly induces DNA damage and triggers the DDR. Surprisingly, IM treatment on PAK6 knockdown cells reversed p21 protein expression induction by PAK6 alone. This could be attributed to the dynamic relationship between senescent and apoptotic cell fates simultaneously induced by PAK6 knockdown. While traditionally viewed as distinct processes, emerging evidence suggests that senescent cells can transition to apoptosis under prolonged stress conditions such as persistent DDR signals [36,42,43,44]. Thus, it may also be fruitful to study the combination of senolytic drugs with PAK6 inhibition and TKI treatment for a more robust targeted therapeutic strategy [45].

## 4. Materials and Methods

### 4.1. Cell Lines

The K562 human CML cell line originated from a 53-year-old female CML blast crisis patient with no BCR::ABL1 kinase mutations. IM-resistant K562 (IMR) cells, a generous gift from Dr. A. Turhan (University of Poitiers, Poitiers, France), were generated by sequentially treating K562 cells with increasing doses of IM and selecting resistant clones that did not harbor BCR::ABL1 kinase mutations.

### 4.2. Primary Patient Samples

Heparin-anticoagulated peripheral blood (PB) or bone marrow (BM) cells were obtained from 23 CML patients (Table S1) and 11 healthy adult donors from the Hematology Cell Bank of BC. Patient samples in this study were retrospectively characterized as either IM-responders or IM-nonresponders. IM-responder characterization was defined by the European Leukemia Net Treatment guidelines and required patients to have achieved at least a 3-log reduction in BCR::ABL1 transcript levels after 12 months of treatment (MMR) and/or show no BCR::ABL1^+^ status after cytogenetic analysis after 6 months of therapy (CCyR) [46,47,48]. Those who did not meet the criteria were considered IM-nonresponders. The mononuclear cells were isolated from the samples using Ficoll-Paque density gradient separation, and CD34^+^ stem/progenitor cells were enriched using the EasySep™ Human CD34 Positive Selection Kit (STEMCELL Technologies, Vancouver, BC, Canada). Verification of stem/progenitor cell purity was performed by staining the cells for the CD34 surface marker with an anti-human CD34 antibody conjugated to allophycocyanin (APC) (BD Biosciences, Mississauga, ON, Canada) and then assessing at least 85% of cells with CD34^+^ expression by FACS analysis.

### 4.3. Small-Molecule Inhibitors

IM (Novartis, Montreal, QC, Canada) and PF-3758309 (Pfizer, Kirkland, QC, Canada) were reconstituted in 10 mM water (IM) or dimethyl sulfoxide (PF-3758309) and stored at −20 °C.

### 4.4. Lentiviral Transfection of PAK6 shRNA Constructs

Two PAK6-coding-domain-targeting shRNA sequences were cloned into a pLKO.1-puro vector under a U6 promoter and obtained as liquid cultures from MilliporeSigma, Oakville, ON, Canada. The first shRNA, shPAK6 1, contained the sense strand CATCCAGAAGTTGTCAGTCAT and the antisense strand ATGACTGACAACTTCTGGATG, whereas for shPAK6 2, the sense strand was CCCAAAGAAGGCAAGTTTGTG, and the antisense strand was CACAAACTTGCCTTCTTTGGGT (Figure S1A). Following the manufacturer’s protocol, the plasmid DNA containing these shRNA sequences was isolated using the PureLink™ HiPure Plasmid Filter Maxiprep Kit (ThermoFisher, Burnaby, BC, Canada). The purified DNA was stored at −20 °C until use.

HEK 293T cells were plated at a density of 5.5 × 10^6^ cells per 10 cm tissue culture plate in Dulbecco’s Modified Eagle Medium (DMEM) supplemented with 10% FBS and 1% glutamine, incubated at 37 °C with 5% CO_2_ [6,12]. After 24 h, the culture medium was replaced with fresh DMEM 4 h before transfection. For each construct, two solutions were prepared. Solution A included construct DNA, ΔR, packaging constructs, and the envelope construct VSVG in Opti-MEM. Solution B contained PEI in Opti-MEM. Solution B was added dropwise to Solution A and incubated at room temperature for 20 min. Subsequently, the DNA-PEI mixture was added to each plate of 293T cells and incubated for 48 h at 37 °C with 5% CO_2_. The culture medium containing the virus was then collected and filtered through a 0.45 μM syringe filter to remove cellular debris. The filtered virus-containing medium was subjected to ultracentrifugation at 2.5 × 10^3^ rpm for 90 min at 4 °C. The supernatant was discarded, and the virus pellet was resuspended in Iscove’s medium with 5% DNase with light agitation for 1 h at room temperature. The virus was then stored at −80 °C until needed.

### 4.5. Lentiviral-Mediated PAK6 Knockdown in CML Cells

K562 and K562-resistant (IMR) cells (3 × 10^5^) were seeded in 24-well plates with 400 μL of RPMI-1640 medium and then transferred to the Biosafety Level 3 facility for transduction. Protamine sulfate was added to each well, along with the scrambled vector control or PAK6 knockdown vector. The cells were incubated at 37 °C with 5% CO_2_ for 16 h, followed by three washes with PBS, then recovered in complete RPMI-1640 medium for at least 24 h. For cells transduced with the PAK6-coding-domain-targeting shRNA, puromycin was added to a final concentration of 2.5 μg/mL. The medium was refreshed every 48 h for at least 5 days to ensure optimal knockdown efficiency. In primary samples, 2 × 10^5^ CD34^+^ CML cells were pre-stimulated in 300 μL of serum-free medium containing growth factors in 24-well plates for 16 h and then similarly transduced with PAK6-coding-domain-targeting shRNA lentiviral particles for 6 h (Supplementary Figure S1A). The cells were then washed and allowed to recover for 48 h before puromycin selection (Supplementary Figure S1A).

### 4.6. Trypan Blue Viability Assay

Cell lines were seeded at a density of 1 × 10^5^ cells/mL in 12-well plates containing complete RPMI-1640 medium. PF-3758309 was administered either alone or in combination with IM for 48 h. Following treatment, cell viability was assessed using a Neubauer hemocytometer after adding trypan blue and PBS. Cells transduced with shRNA were allowed to recover in complete RPMI-1640 medium for at least 48 h before seeding. For IC50 measurements, cells were treated with PF-3758309 at increasing concentrations (0 nM, 1 nM, 10 nM, 100 nM, 1000 nM). Viability data were plotted, and the IC50 curve and values were calculated using the Dose-Response—Inhibition Non-Linear Fit function in GraphPad PRISM 8 version.

### 4.7. PI-Annexin V Apoptosis Assay

Cells that remained after the viability assay at 48 h post-treatment were stained with Annexin V APC and Propidium Iodide (PI) using the Annexin V Apoptosis Detection Kit APC (eBioscience, San Diego, CA, USA) to assess apoptosis according to the manufacturer’s instructions. The prepared cells were kept on ice and analyzed by flow cytometry with FACSCalibur (BD Bioscience, Mississauga, ON, Canada) and FlowJo^®^ v10 software. Apoptotic populations were identified based on Annexin V^+^ staining, distinguishing early apoptosis (Annexin V^+^/PI^−^) and late apoptosis (Annexin V^+^/PI^+^) populations.

### 4.8. CFC Assay

For colony-forming cell (CFC) assays, 3000 CD34^+^ CML cells were mixed in MethoCult H4230 (STEMCELL Technologies, Vancouver, BC, Canada) containing growth factor cocktails (IL-6, IL-3, GM-CSF, G-CSF). The MethoCult mixture was aliquoted into polystyrene round-bottom tubes using a syringe with a blunt-end needle, with or without IM. The mixture was vortexed and plated onto culture plates, followed by incubation at 37 °C with 5% CO_2_. After 14 days, colonies were counted and expressed as CFC output per input cell for specific colony types (GEMM, BFU-E, and CFU-GM).

### 4.9. Cell Cycle Assay

Cells were washed three times with PBS and resuspended to a concentration of approximately 3 × 10^6^ cells/mL. Ice-cold 100% ethanol was added to fix the cells, which were left overnight at 4 °C. The ethanol was then removed, and the cells were washed with PBS before being resuspended in a solution containing propidium iodide (PI), RNase, and Triton X-100. The cells were stained for 30 min in the dark, washed, and resuspended in ice-cold PBS prior to analysis by flow cytometry using the FACSCalibur (BD Bioscience, Mississauga, ON, Canada) and FlowJo^®^ v10 software.

### 4.10. Senescence-Associated β-Galactosidase Staining

Senescence-associated β-Galactosidase chromogenic and FACS staining were conducted following standard protocols [49]. For chromogenic staining, cells were adhered to Poly-L-Lysine-coated slides for 20 min at 37 °C. The cells were then fixed and stained for β-Galactosidase using the Senescence-Associated β-Galactosidase Staining Kit (Cell Signaling, Danvers, MA, USA) per the manufacturer’s instructions. Slides were examined, and images were captured using a Nikon C1 confocal microscope with a 40× objective lens. For FACS detection, cells were seeded into 6-well plates, with 80 nM doxorubicin-treated cells as a positive control. After 48 h, a portion of the cells was used to determine the total cell number using a hemocytometer. The remaining cells were pre-treated with 100 nM bafilomycin A1 for 1 h at 37 °C, 5% CO_2_. Cells were then incubated with 33 μM 5-Dodecanoylaminofluorescein Di-β-D-Galactopyranoside (C12FDG) for 30 min at 37 °C, 5% CO_2_, washed with PBS at room temperature, and resuspended in 400 μL of ice-cold PBS. The cell suspensions were immediately analyzed using a FACSCalibur flow cytometer and FlowJo^®^ v10 software.

### 4.11. Wright-Giemsa Staining

Cells were placed on slides using a cytospin and stained with the Hematology Stain Pack Modified Wright’s Stain (VWR) using the Hematek^®^ Slider Stainer instrument, following the manufacturer’s instructions. The slides were then examined, and images were captured using a Nikon C1 confocal microscope with a 20× objective lens.

### 4.12. RNA Extraction

RNA was extracted using TRIzol (Life Technologies, Toronto, ON, Canada) following the manufacturer’s standard protocol. The RNA pellet was resuspended in UltraPure DNase/RNase-free water (Life Technologies, Toronto, ON, Canada) and quantified for concentration and purity using a NanoDrop spectrophotometer (ThermoFisher Scientific, Burnaby, BC, Canada). The RNA was then stored at −20 °C until further use.

### 4.13. Quantitative Real-Time PCR

A total of 500 ng of RNA was reverse transcribed into cDNA using the STEMscript cDNA Synthesis Kit with Random Primers (StemCell Technologies, Vancouver, BC, Canada) according to the manufacturer’s instructions. The cDNA was then combined with oligo primers for PAK6, MDM2, p21, p27, MMP3, and B2M, along with RNase-free water and 2X SYBR Green PCR Master Mix (Life Technologies, Toronto, ON, Canada) in a 96-well plate for qRT-PCR analysis using the 7500 Real-Time PCR System (Applied Biosystems, Foster City, CA, USA). Primers used for qRT-PCR are listed in Table S2.

### 4.14. Cell Lysis and Protein Quantification

Cell pellets were washed twice with phosphate-buffered saline (PBS) (Life Technologies, Toronto, ON, Canada), flash frozen, and lysed with a homemade lysis buffer consisting of 90% phosphorylation solubilizing buffer (PSB), 10% NP-40, 0.1% sodium dodecyl sulfate (SDS), 0.5% 200 mM phenylmethylsulfonyl fluoride (PMSF), and 0.5% protease inhibitor cocktail for 20 min at 4 °C. The lysed pellets were then centrifuged at 14,000 rpm for 20 min at 4 °C to separate the cell debris. The supernatant was collected, and protein concentration was measured using the Bradford protein assay with absorbance read at 570 nm on a spectrophotometer, using the lysis buffer as a control.

### 4.15. Western Blot Analysis

Protein lysates were mixed with 2× Laemmli loading buffer and ddH2O, then heated at 90 °C for 10 min for denaturation. Samples were run on 10% SDS-PAGE gels and transferred to PVDF membranes for 1.5 h on ice. Membranes were blocked with 5% skim milk for 30 min, then incubated overnight at 4 °C with primary antibodies in TBST (5% BSA for phospho-proteins, skim milk for total proteins). After washing with TBST, secondary antibodies were added for 1 h at room temperature. The membranes were washed, treated with ECL reagent, and imaged using the ChemiDoc MP system (Bio-Rad, Mississauga, ON). Primary antibodies included PAK6 (1:1000, Bio-Rad), β-Actin (1:500), MDM2 (1:200, Santa Cruz, Dallas, TX, USA), p21, γH2AX, p27, MMP3 (1:2000, Cell Signaling, Danvers, MA, USA), 4G10 (1:5000), pPAK6 (1:500), ATM and ATR (1:1000, Cell Signaling, Danvers, MA, USA), and pATR and pATM (1:500, Cell Signaling, Danvers, MA, USA). Protein expression was quantified using FIJI ImageJ2 software, normalizing to β-Actin.

### 4.16. Bioinformatic Analysis

Cancer cell lines and drug activity were profiled using CellMinerDB (version 1.2) and the Cancer Therapeutics Research Portal Version 2 (CTRPv2) database to perform univariate analysis and generate correlation plots between drug sensitivity and gene expression [23,32]. Gene set enrichment analysis (GSEA) was conducted with the Enrichr tool, cross-referencing PAK6 substrate gene sets from the Kinase Library Serine Threonine Kinome Atlas, PhosphositePlus, and Pathway Commons Protein-Protein against the Molecular Signatures Database (MSigDB, 2024.1) [25,26,27,50]. The GSE14671 microarray dataset from the Gene Expression Omnibus (GEO) was analyzed to compare gene expression in CD34^+^ cells between 12 IM-nonresponders and 24 IM-responders [20]. Samples were collected at diagnosis, with response status determined 12 months post-IM therapy [20]. The dataset used the Affymetrix Human Genome U133 Plus 2.0 Array system, and gene expression was determined via the median of robust multi-array average (RMA) gene probe values.

### 4.17. Statistical Analysis

Results are presented as the mean ± standard error of the mean from at least three independent experiments. Group differences were assessed using the 2-tailed Student’s *t*-test or one-way ANOVA for multiple comparisons.

## 5. Conclusions

Overall, our findings indicate that PAK6 knockdown induces G2-M cell cycle arrest in CML cells, suggesting that PAK6 facilitates leukemia growth by dysregulating cell cycle checkpoints. Additionally, PAK6 knockdown mediates the MDM2-p21, contributing to cell cycle arrest and senescence. The observed increase in phosphorylated ATM/ATR and senescence markers like p21, p27, MMP3, and γH2AX further supports the role of PAK6 in regulating DDR-associated senescence. Thus, our study provides promising evidence that targeting PAK6, either through pharmacological inhibitors or genetic suppression, could offer a new therapeutic strategy in combination with TKIs or other new drugs for developing effective therapies for clinical applications.

## Data Availability

Data are contained within the article and Supplementary Materials.

## Supplementary Materials

The following supporting information can be downloaded at: https://www.mdpi.com/article/10.3390/ijms26136533/s1.
