# Supplementary material for: Identification of a PAK6-Mediated MDM2/p21 Axis That Modulates Survival and Cell Cycle Control of Drug-Resistant Stem/Progenitor Cells in Chronic Myeloid Leukemia
Source: Int J Mol Sci. 2025 Jul 7;26(13):6533. doi: 10.3390/ijms26136533 (PMC12250115; doi:10.3390/ijms26136533)
Supplement: Supplementary file 1 [file ijms-26-06533-s001.zip › ijms-3716424-supplementary.pdf]

Supplementary Table S1: **Overview of all primary CML samples used in this study**

Patient code	Sex	Age	Sokal score	White blood cell count ($x10^6/L$)	Response Status*
**1**	M	32	High	N/A	NR
**2**	M	36	Intermediate	156.1	NR
**3**	M	38	Low	144.2	NR
**4**	M	55	Intermediate	227.7	NR
**5**	M	39	Low	46.8	NR
**6**	F	52	High	49.4	NR
**7**	F	60	Intermediate	33.4	NR
**8**	M	58	N/A	N/A	NR
**9**	M	21	High	184.6	NR
**10**	F	55	Intermediate	271.5	NR
**11**	F	60	High	185.5	NR
**12**	F	59	Intermediate	75.0	R
**13**	F	55	Intermediate	77.8	R
**14**	M	53	High	246.8	R
**15**	M	21	Low	84.6	R
**16**	F	34	Low	230.4	R
**17**	M	47	Low	40.5	R
**18**	M	34	Low	41.1	R
**19**	M	38	Intermediate	116.5	R
**20**	M	69	High	166.4	R
**21**	M	68	N/A	176.2	R
**22**	F	67	High	303.9	R
**23**	M	46	Low	249.3	R

*Patient response status was classified into responders (R) and IM-nonresponders (NR) based on the 2013 and 2020 TKI Failure European Leukemia Net guidelines. Responders achieved a 1-log fold reduction of BCR::ABL1 transcripts by at least 6 months, while IM-nonresponders did not achieve this threshold of response to these response criteria. N/A=data not available.

Supplementary Table S2: List of oligo primers used for qRT-PCR analysis

Gene name	Forward primer	Reverse primer
**PAK6**	GGCCAGAGACAGGAATGTAAG	TTCGCCTTCTCTCCCAGATA
**MDM2**	AGTTGCGCTTTATGGGTGGA	TCCAAGTGCTGGTGCTTTCA
**p21**	CGACTGTGATGCGCTAATGG	CGTTTTCGACCCTGAGAG
**p27**	CTGATGCTGTTGCTCGGTTA	TGCAGACTCTGGGACATCTG
**MMP3**	CACTCACAGACCTGACTCGGTT	AAGCAGGATCACAGTTGGCTGG

Supplementary Table S3: Lists of substrates and common interactors of PAK6

Substrates of PAK6			Common interactors of PAK6
MDM2	MRVI1	GTF3C1	HSP90AA1
MAP3K2	KIF2C	MAP1LC3A	LIMK1
SCAF11	RLTPR	FAM83H	NEK6
PPP1R16B	ANKRD33B	SPAST	ESR1
PLEKHA6	CDCA2	GRK7	CDC42
PRPF4B	IRS1	XIRP2	TPD52L1
MYO7B	RIF1	DRD2	MAPK14
CDC25B	CDCA8	FAM83E	AR
DEPDC1B	WDR43	FAM83B	PKM
ASPHD1	PRKDC	RHBDF2	ACTA2
C6ORF25	GATA3	PLEKHG2	ACTA1
MSH6	RASSF1	PLEKHG3	ACTBL2
WDR81	KAT5	ATRX	CDK1
ZC3H11A	HIVEP1	STXBP5L	LNX1
PKP3	PPP1R10	SCAF1	RHOU
ALPK3	TP53BP2	MYO1C	MDM2
LIN9	CCDC120	FAM131B	APP
PANK2	PPP1R12A	POLR1E	ROCK1
RALA	MYBPC3	KCTD15	HSP90AB1
TESK2	PPP1R12B	SPAG1	LRRK1
FAM214B	STRADA	PLEKHG1	SEMA3B
RBM15B	NFATC2	LLGL2	LRRK2
RAD54L	PALB2	ZNF212	ACTG1
SRBD1	SGOL1	PPP1R13B	STMN4
OSBPL5	ETV6	PFAS	ACTC1
KIF23	TMEM217	PKD1	ACTG2
DGKZ	PDE3B	MKI67	ACTB
CCDC88C	RAPGEF1	PIK3C2B	SNX2
DMXL1	PLEC	GLI1	STMN1
RGCC	TNFRSF11A	TRAK1	YWHAQ
WNK1	RASGRF1	SETD2	AKT1
WNK2	MAST3		RAC1
GOLGB1	MAST2		RAC2
WNK4	UBR5		PACSIN1
CDC42EP4	AEBP2		MAP2K6
	GTF3C1		

A

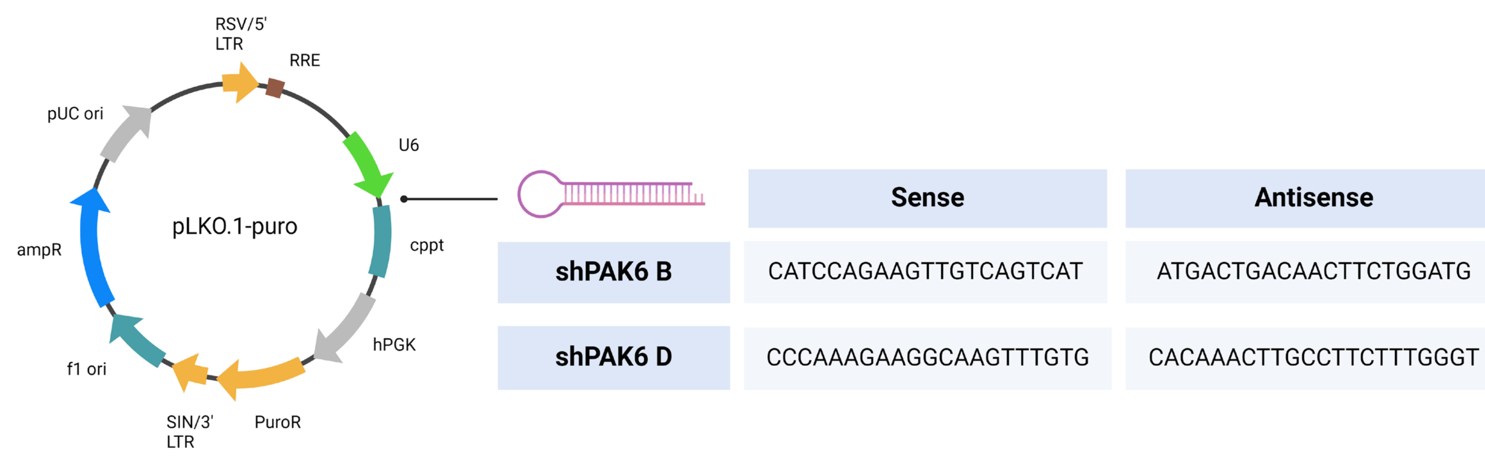

	Sense	Antisense
**shPAK6 B**	CATCCAGAAGTTGTCAGTCAT	ATGACTGACAACTTCTGGATG
**shPAK6 D**	CCCAAAGAAGGCAAGTTTGTG	CACAAACTTGCCTTCTTTGGGT

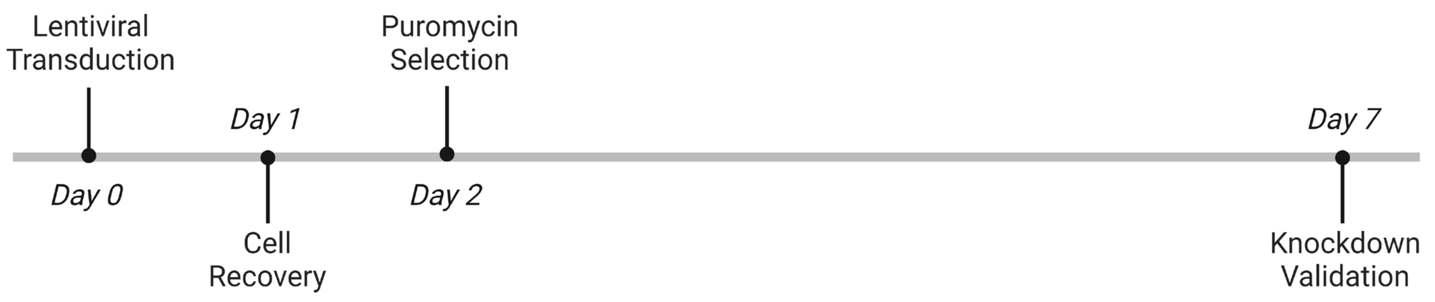

B

PAK6 Substrates	Molecular Weight(DA)	Residual	Validation Method
AR	99,188	S579-p	In Vivo, In Vitro
CTBP1	47,535	S100-p	In Vitro
CTBP1	47,535	S158-p	In Vitro
CTBP2	48,945	S164-p	In Vitro
ICAP1	21,782	S10-p	In Vivo
LIMK1	72,585	T508-p	In Vivo, In Vitro
MDM2	55,233	T158-p	In Vivo, In Vitro
MDM2	55,233	S186-p	In Vivo, In Vitro
PACSIN1	50,966	S346-p	In Vivo

C

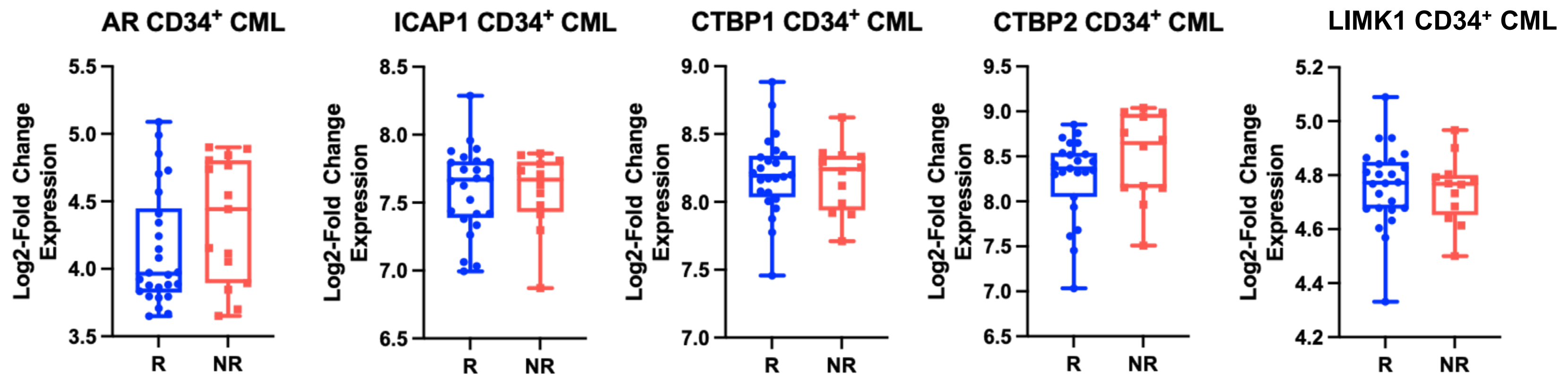

Supplementary Figure S1: **Lentiviral-mediated PAK6 knockdown in CML cells and identification of PAK6 substrates**. **(A)** Cells were transduced with two PAK6 coding-domain targeting shRNA constructs or a scrambled control vector, followed by puromycin selection to ensure knockdown efficiency. Schematic representation of lentiviral-mediated transduction of PAK6-targeting shRNA in K562, K562-resistant (IMR), and primary $CD34^+$ CML cells. (**B**). Lists of PAK6 substrates using the PhosphoSitePlus platform. (**C**) Analysis of the GSE14671 microarray dataset comparing gene expression in $CD34^+$ cells from IM-responders and IM-nonresponders revealed no significant differences in the expression levels of other PAK6 substrates besides MDM2.
